# Asynchronous visual event-based time-to-contact

**DOI:** 10.3389/fnins.2014.00009

**Published:** 2014-02-07

**Authors:** Xavier Clady, Charles Clercq, Sio-Hoi Ieng, Fouzhan Houseini, Marco Randazzo, Lorenzo Natale, Chiara Bartolozzi, Ryad Benosman

**Affiliations:** ^1^Vision Institute, Universitée Pierre et Marie Curie, UMR S968 Inserm, UPMC, CNRS UMR 7210, CHNO des Quinze-VingtsParis, France; ^2^iCub Facility, Istituto Italiano di TecnologiaGenova, Italia

**Keywords:** neuromophic vision, event-based computation, time to contact, robotics, computer vision

## Abstract

Reliable and fast sensing of the environment is a fundamental requirement for autonomous mobile robotic platforms. Unfortunately, the frame-based acquisition paradigm at the basis of main stream artificial perceptive systems is limited by low temporal dynamics and redundant data flow, leading to high computational costs. Hence, conventional sensing and relative computation are obviously incompatible with the design of high speed sensor-based reactive control for mobile applications, that pose strict limits on energy consumption and computational load. This paper introduces a fast obstacle avoidance method based on the output of an asynchronous event-based time encoded imaging sensor. The proposed method relies on an event-based Time To Contact (TTC) computation based on visual event-based motion flows. The approach is event-based in the sense that every incoming event adds to the computation process thus allowing fast avoidance responses. The method is validated indoor on a mobile robot, comparing the event-based TTC with a laser range finder TTC, showing that event-based sensing offers new perspectives for mobile robotics sensing.

## 1. Introduction

A fundamental navigation task for autonomous mobile robots is to detect and avoid obstacles in their path. This paper introduces a full methodology for the event-based computation of Time To Contact (TTC) for obstacle avoidance, using an asynchronous event-based sensor.

Sensors such as ultrasonic sensors, laser range finders or infrared sensors are often mounted on-board of robotic platforms in order to provide distance to obstacles. Such active devices are used to measure signals transmitted by the sensor and reflected by the obstacle(s). Their performance is essentially dependent on how the transmitted energy (ultrasonic waves, light,…) interacts with the environment Everett (1995); Ge (2010).

These sensors have limitations. In the case of ultrasonic sensors, corners and oblique surfaces, or even temperature variations can provide artifacts in the measurements. Infrared-based sensors (including recently emerged Time-Of-Light or RGB-D cameras) are sensitive to sunlight and can fail if the obstacle absorbs the signal. Laser range finder readings may also be erroneous because of specular reflections; additionally, the potential problems of eye-safety limit the use of many laser sensors to environments where humans are not present. In addition, most of the sensors have restrictions in terms of field-of-view and/or spatial resolution, requiring a mechanical scanning system or a network of several sensors. This leads to severe restrictions in terms of temporal responsiveness and computational load.

Vision can potentially overcome many of these restrictions; visual sensors often provide better resolution, wider range at faster rates than active scanning sensors. Their capacity to detect the natural light reflected by the objects or the surrounding areas paves the way to biological-inspired approaches.

Several navigation strategies using vision have been proposed, the most common consist of extracting depth information from visual information. Stereo-vision techniques can also produce accurate depth maps if the stability of the calibration parameters and a relative sufficient inter-camera distance can be ensured. However, these are strong requirements for high-speed and small robots. Another class of algorithms (Lorigo et al., 1997; Ulrich and Nourbakhsh, 2000), is based on color or texture segmentation of the ground plane. Even if this approach works on a single image, it requires the assumption that the robot is operating on a flat and uni-colored/textured surface and all objects have their bases on the ground.

Another extensively studied strategy is based on the evaluation of the TTC, noted τ. This measure, introduced by Lee (1976), corresponds to the time that would elapse before the robot reaches an obstacle if the current relative motion between the robot and the obstacle itself were to continue without change. As the robot can navigate through the environment following a trajectory decomposed into straight lines (which is a classic and efficient strategy for autonomous robots in most environments), a general definition of TTC can be expressed as follows:

(1) $$\tau =-\frac{Z}{\frac{dZ}{dt}}$$

where *Z* is the distance between the camera and the obstacle, and $\frac{dZ}{dt}$ corresponds to the relative speed.

The Time-to-contact can be computed considering only visual information, without extracting relative depth information and speed, as demonstrated by Camus (1995) (see Section 3.2). Its computation has the advantage to work with a single camera, without camera calibration or binding assumptions about the environment. Several techniques for the measure of TTC have been proposed. In Negre et al. (2006); Alyena et al. (2009), it is approximated measuring the local scale change of the obstacle, under the assumption that the obstacle is planar and parallel to the image plane. This approach requires either to precisely segment the obstacle in the image or to compute complex features in multi-scales representation of the image. Most studied methods of TTC rely on the estimation of optical flow. Optical flow conveys all necessary information from the environment Gibson (1978), but its estimation on natural scenes is well-known to be a difficult problem. Existing techniques are computationally expensive and are mostly used off line (Negahdaripour and Ganesan, 1992; Horn et al., 2007). Real-time implementations, using gradient-based, feature matching-based (Tomasi and Shi, 1994) or differential ones, do not deal with large displacements. Multi-scale process, as proposed by Weber and Malik (1995), can manage with this limitation, at the cost of computing time and hardware memory to store and process frames at different scales and timings.

Rind and Simmons (1999) proposed a bio-inspired neural network modeling the lobula giant movement detector (LGMD), a visual part of the optic lobe of the locust that responds most strongly to approaching objects. In order to process the frames provided by a conventional camera, existing implementations proposed by Blanchard et al. (2000) and Yue and Rind (2006) required a distributed computing environment (three PCs connected via ethernet). Another promising approach consists in VLSI architecture implementing functional models of similar neural networks, but it will require huge investments to go beyond the single proof of concept, such as the 1-D architecture of 25 pixels proposed by Indiveri (1998) modeling locust descending contralateral movement detector (DCMD) neurons. The hardware systems constructed in Manchester and Heidelberg, and described respectively by Bruderle et al. (2011) and Furber et al. (2012), could be an answer to this issue.

Globally, most of these approaches suffer from the limitations imposed by frame-based acquisition of visual information in the conventional cameras, that output large and redundant data flow, at a relative low temporal frequency. Most of the calculations are operated on uninformative parts of the images, or are dedicated to compensate for the lack of temporal precision. Existing implementations are often a trade off between accuracy and efficiency and are restricted to mobile robots moving relatively slowly. For example, Low and Wyeth (2005) and Guzel and Bicker (2010) present experiments on the navigation of a wheeled mobile robotic platform using optical flow based TTC computation applied with an embedded conventional camera. Their softwares run at approximatively 5 Hz and the maximal speed of the mobile robot is limited to 0.2 m/s.

In this perspective, free-frame acquisition of the neuromorphic cameras (Guo et al., 2007; Lichtsteiner et al., 2008; Lenero-Bardallo et al., 2011; Posch et al., 2011), can introduce significant improvements in robotic applications. The operation of such sensors is based on independent pixels that asynchronously collect and send their own data, when the processed signal exceeds a tunable threshold. The resulting compressed stream of events includes the spatial location of active pixels and an accurate time stamping at which a given signal change occurs. Events can be processed locally while encoding the additional temporal dynamics of the scene.

This article presents an event-based methodology to measure the TTC from the events stream provided by a neuromorphic vision sensor mounted on a wheeled robotic platform. The TTC is computed and then updated for each incoming event, minimizing the computational load of the robot. The performance of the developed event-based TTC is compared with a laser range finder, showing that event-driven sensing and computation, with their sub-microsecond temporal resolution and the inherent redundancy suppression, are a promising solution to vision-based technology for high-speed robots.

In the following we briefly introduce the used neuromorphic vision sensor (Section 2, describe the event-based approach proposed to compute the TTC (Section 3) and present experimental results validating the accuracy and the robustness of the proposed technique on a mobile robots moving in an indoor environment (Section 4).

## 2. Time encoded imaging

Biomimetic, event-based cameras are a novel type of vision devices that—like their biological counterparts—are driven by “events” happening within the scene, and not by artificially created timing and control signals (i.e., frame clock of conventional image sensors) that have no relation whatsoever with the source of the visual information. Over the past few years, a variety of these event-based devices, reviewed in Delbruck et al. (2010), have been implemented, including temporal contrast vision sensors that are sensitive to relative light intensity change, gradient-based sensors sensitive to static edges, edge-orientation sensitive devices and optical-flow sensors. Most of these vision sensors encode visual information about the scene in the form of asynchronous address events (AER) Boahen (2000)using time rather than voltage, charge or current.

The ATIS (“Asynchronous Time-based Image Sensor”) used in this work is a time-domain encoding image sensor with QVGA resolution Posch et al. (2011). It contains an array of fully autonomous pixels that combine an illuminance change detector circuit and a conditional exposure measurement block.

**Figure 1:**
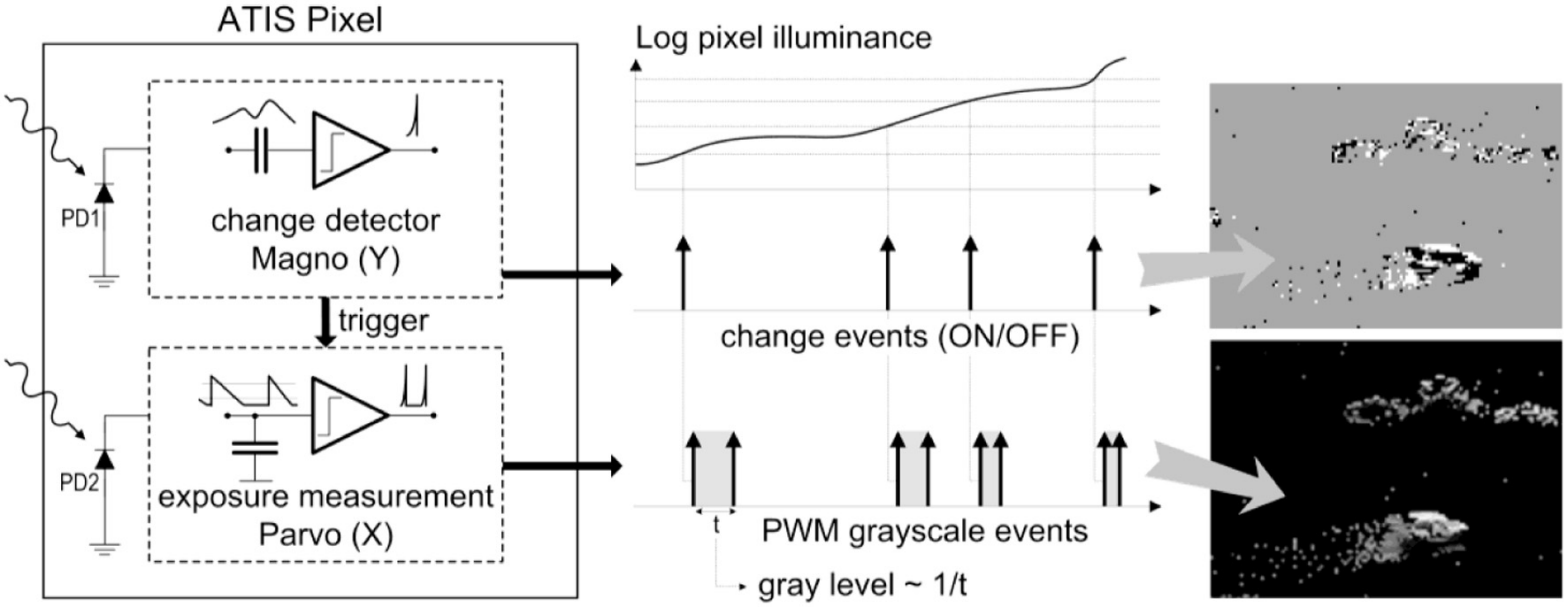
**Functional diagram of an ATIS pixel Posch (2010)**. Two types of asynchronous events, encoding change and brightness information, are generated and transmitted individually by each pixel in the imaging array.

As shown in the functional diagram of the ATIS pixel in Figure 1, the change detector individually and asynchronously initiates the measurement of an exposure/gray scale value only if a brightness change of a certain magnitude has been detected in the field-of-view of the respective pixel. The exposure measurement circuit encodes the absolute instantaneous pixel illuminance into the timing of asynchronous event pulses, more precisely into inter-event intervals.

Since the ATIS is not clocked, the timing of events can be conveyed with a very accurate temporal resolution in the order of microseconds. The time-domain encoding of the intensity information automatically optimizes the exposure time separately for each pixel instead of imposing a fixed integration time for the entire array, resulting in an exceptionally high dynamic range and an improved signal to noise ratio. The pixel-individual change detector driven operation yields almost ideal temporal redundancy suppression, resulting in a sparse encoding of the image data.

**Figure 2:**
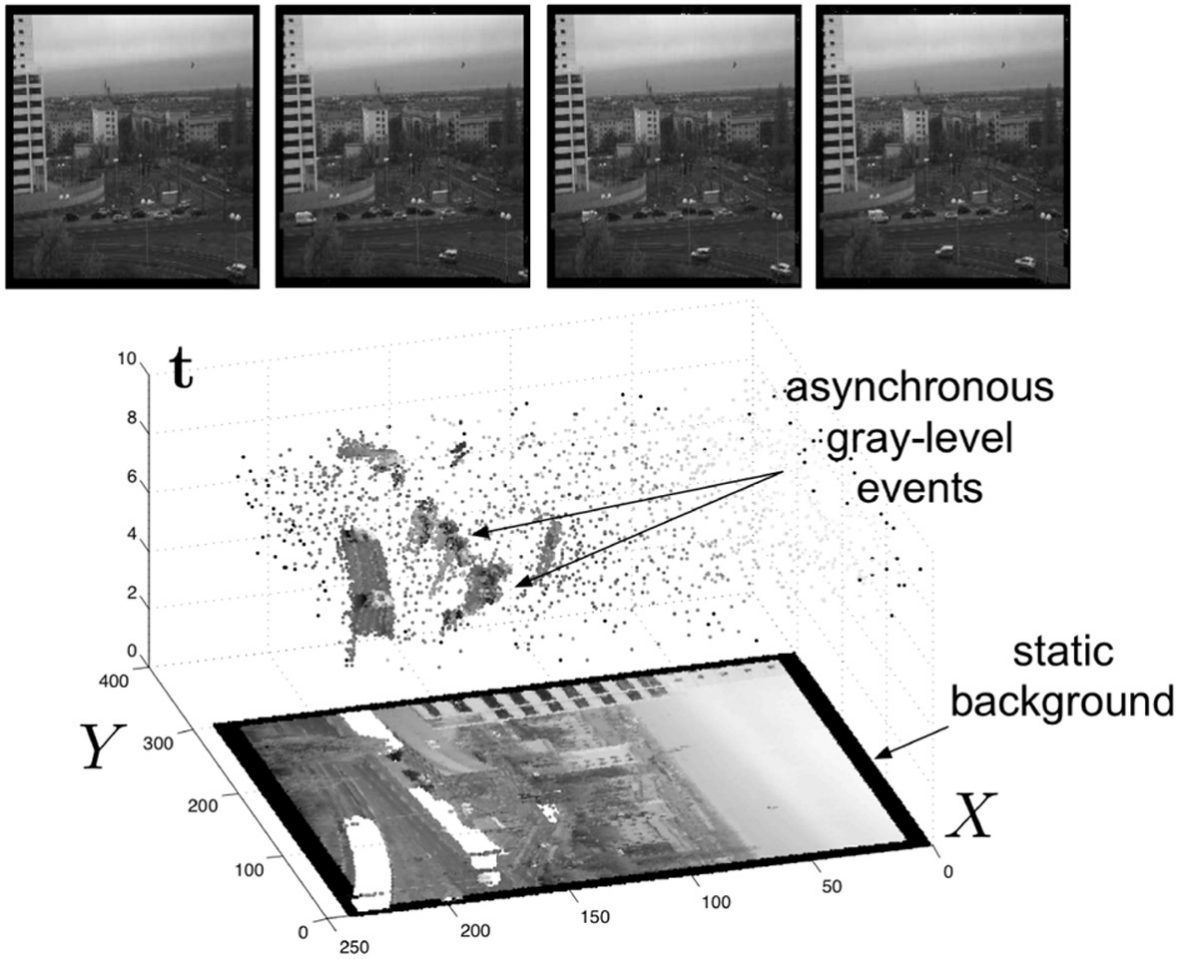
**Lower part The spatio-temporal space of imaging events: static objects and scene background are acquired first**. Then, dynamic objects trigger pixel-individual, asynchronous gray-level events after each change. Frames are absent from this acquisition process. Samples of generated images from the presented spatio-temporal space are shown in the upper part of the figure.

Figure 2 shows the general principle of asynchronous imaging in a spatio-temporal representation. Frames are absent from this acquisition process. They can however be reconstructed, when needed, at frequencies limited only by the temporal resolution of the pixel circuits (up to hundreds of kiloframes per second) (Figure 2 top). Static objects and background information, if required, can be recorded as a snapshot at the start of an acquisition. And henceforward moving objects in the visual scene describe a spatio-temporal surface at very high temporal resolution (Figure 2 bottom).

## 3. Event-based TTC computation

### 3.1. Event-based visual motion flow

The stream of events from the silicon retina can be mathematically defined as follows: let *e*(**p**, t) = (**p**, t)^*T*^ a triplet giving the position **p** = (*x, y*)^*T*^ and the time *t* of an event. We can then define locally the function Σ_*e*_ that maps to each **p**, the time *t*:

(2) $$\begin{array}{c} \begin{array}{l} \;\Sigma_{e}:{\mathbb{N}}^{2}\to \mathbb{R}\\ \text{p}\mapsto \Sigma_{e}(\text{p})=t \end{array} \end{array}$$

Time being an increasing function, Σ_*e*_ is a monotonically increasing surface in the direction of the motion.

**Figure 3:**
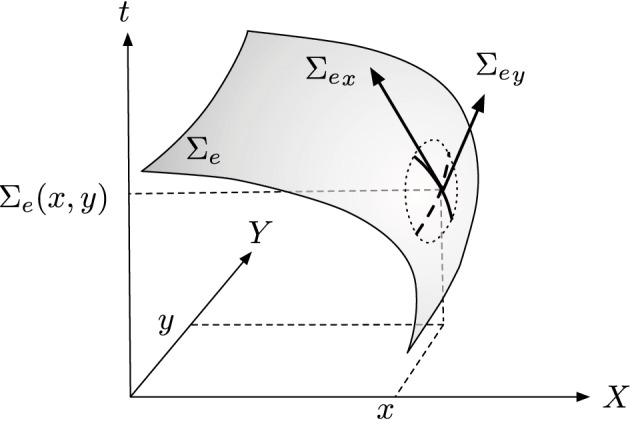
**General principle of visual flow computation, the surface of active events Σ_*e*_ is derived to provide an estimation of orientation and amplitude of motion**.

We then set the first partial derivatives with respect to the parameters as: $\Sigma_{e_{x}}=\frac{\partial \Sigma_{e}}{\partial x}$ and $\Sigma_{e_{y}}=\frac{\partial \Sigma_{e}}{\partial y}$ (see Figure 3). We can then write Σ_*e*_ as:

(3) $$\Sigma_{e}(p+\Delta p)=\Sigma_{e}(p)+\nabla \Sigma_{e}^{T}\Delta p+o(||\Delta p||)$$

with $\nabla \Sigma_{e}={\left(\frac{\partial \Sigma_{e}}{\partial x},\frac{\partial \Sigma_{e}}{\partial y}\right)}^{T}$.

The partial functions of Σ_*e*_ are functions of a single variable, whether *x* or *y*. Time being a strictly increasing function, Σ_*e*_ is a nonzero derivatives surface at any point. It is then possible to use the inverse function theorem to write around a location **p** = (*x, y*)^*T*^:

(4) $$\begin{array}{l} {\left(\frac{\partial \Sigma_{e}}{\partial x}(x,y_{0}),\frac{\partial \Sigma_{e}}{\partial y}(x_{0},y)\right)}^{T}={\left(\frac{\text{d}\Sigma_{e}{|}_{y=y_{0}}}{\text{d}x}(x),\frac{\text{d}\Sigma_{e}{|}_{x=x_{0}}}{\text{d}y}(y)\right)}^{T}\\ \;={\left(\frac{1}{v_{n}{}_{x}(x,y_{0})},\frac{1}{v_{n}{}_{y}(x_{0},y)}\right)}^{T} \end{array}$$

$\Sigma_{e}{|}_{x=x_{0}}$, $\Sigma_{e}{|}_{y=y_{0}}$ being Σ_*e*_ restricted respectively to *x* = *x*_0_ and *y* = *y*_0_, and **v**_n_(*x, y*) = (*v_nx_, v_ny_*)^*T*^ represents the normal component of the visual motion flow; it is perpendicular to the object boundary (describing the local surface Σ_*e*_).

The gradient of Σ_*e*_ or ∇Σ_*e*_, is then:

(5) $$\nabla \Sigma_{e}(\text{p},t)={\left(\frac{1}{v_{n}{}_{x}(x,y_{0})},\frac{1}{v_{n}{}_{y}(x_{0},y)}\right)}^{T}$$

The vector ∇Σ_*e*_ measures the rate and the direction of change of time with respect to the space, its components are also the inverse of the components of the velocity vector estimated at **p**.

The flow definition given by Equation 5 is sensitive to noise since it consists in estimating the partial derivatives of Σ_*e*_ at each individual event. One way to make the flow estimation robust against noise is to add a regularization process to the estimation. To achieve this, we assume a local velocity constancy. This hypothesis is satisfied in practice for small clusters of events. It is then equivalent to assume Σ_*e*_ being locally planar since its partial spatial derivatives are the inverse of the speed, hence constant velocities produce constant spatial rate of change in Σ_*e*_. Finally, the slope of the fitted plane with respect to the time axis is directly proportional to the motion velocity. The regularization also compensates for absent events in the neighborhood of active events where motion is being computed. The plane fitting provides an approximation of the timing of still non active spatial locations due the non idealities and the asynchronous nature of the sensor. The reader interested in the computation of motion flow can refer to Benosman et al. (2014) for more details. A full characterization of its computational cost is proposed; it shows that the event-based calculation required much less computation time than the frame-based one.

### 3.2. Time-to-contact

Assuming parts of the environment are static, while the camera is moving forward, the motion flow diverges around a point called the focus of expansion (FOE). The visual motion flow field and the corresponding focus of expansion can be used to determine the time-to-contact (TTC) or time-to-collision. If the camera is embedded on an autonomous robot moving with a constant velocity, the TTC can be determined without any knowledge of the distance to be traveled or the velocity the robot is moving.

**Figure 4:**
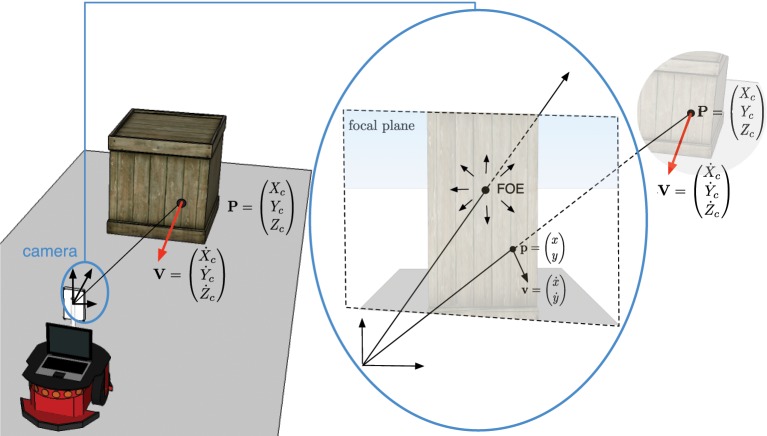
**3D obstacle velocity *V* projected into the camera focal plane as *v***. The dotted letters refer to temporal derivatives of each component.

We assume the obstacle is at **P** = (*X_c_, Y_c_, Z_c_*)^*T*^ in the camera coordinate frame and **p** = (*x, y*)^*T*^ is its projection into the camera's focal plane coordinate frame (see Figure 4). The velocity vector **V** is also projected into the focal plane as **v** = ($\dot{x},\dot{y}$)^*T*^.

By deriving the pinhole model's equations, Camus (1995) demonstrates that, if the coordinates **p***f* = (*x_f_, y_f_*)^*T*^ of the FOE are known, the following relation is satisfied:

(6) $$\begin{array}{l} \;\tau =-\frac{Z_{c}}{{\dot{Z}}_{c}}=\frac{y-y_{f}}{\dot{y}}=\frac{x-x_{f}}{\dot{x}},\text{where}\\ {\dot{Z}}_{c}=\frac{dZ_{c}}{dt},\;\dot{x}=\frac{dx}{dt},\;\dot{y}=\frac{dy}{dt}. \end{array}$$

With our notation, this is equivalent to:

(7) $$\tau (p,t)\text{v}(p,t)=p-p_{f}$$

The TTC is then obtained at pixel **p** according to the relation:

(8) $$\tau (p,t)=\frac{{\text{v}}^{T}(p,t)(p-p_{f})}{||\text{v}(p,t)|{|}^{2}}$$

The TTC as defined is a signed real value because of the scalar product. Its sign refers to the direction of the motion: when τ is positive, the robot is going toward the obstacle and, viceversa, for negative τ it is getting away. This equality shows also that τ can be determined only if the velocity **v** at **p** is known or can be estimated for any **p** at anytime *t*. There is unfortunately no general technique for estimating densely the velocity **v** from the visual information. However, optical flow techniques allow to compute densely the vector field of velocities normal to the edges, noted as **v**_*n*_. The visual flow technique presented in subsection 3.2 is the ideal technique to compute τ, not only because of its event-based formulation, but it is also showing that the normal to the edge component of **v** is sufficient for τ determination. From Equation 7, we apply the scalar product of both end sides with ∇Σ_*e*_:

(9) $$\tau (p,t)\text{v}{\left(p,t\right)}^{T}\nabla \Sigma_{e}(p,t)={\left(p-p_{f}\right)}^{T}\nabla \Sigma_{e}(p,t)$$

Because **v** can be decomposed as the sum of a tangential vector **v**_*t*_, and a normal vector **v**_*n*_, the left end side of Equation 9 simplifies into:

(10) $$\begin{array}{l} \tau {\text{v}}^{T}(p,t)\nabla \Sigma_{e}(p,t)=\tau {\left({\text{v}}_{t}(p,t)+{\text{v}}_{n}(p,t)\right)}^{T}\nabla \Sigma_{e}(p,t)\\ \;=\tau {\text{v}}_{n}^{T}(p,t)\nabla \Sigma_{e}(p,t)=2\tau \end{array}$$

**v**^*T*^_*t*_ ∇ Σ_*e*_ = 0 since the tangential component is orthogonal to ∇Σ_*e*_. Therefore τ is given by:

(11) $$\tau (p,t)=\frac{1}{2}{\left(p-p_{f}\right)}^{T}\nabla \Sigma_{e}(p,t)$$

### 3.3 Focus of expansion

The FOE is the projection of the observer's direction of translation (or heading) on the sensor's image plane. The radial pattern of flows depends only on the observer's heading and is independent of 3D structure, while the magnitude of flow depends on both heading and depth. Thus, in principle, the FOE could be obtained by triangulation of two vectors in a radial flow pattern. However, such a method would be vulnerable to noise. To calculate the FOE, we used the redundancy in the flow pattern to reduce errors.

**Figure 5:**
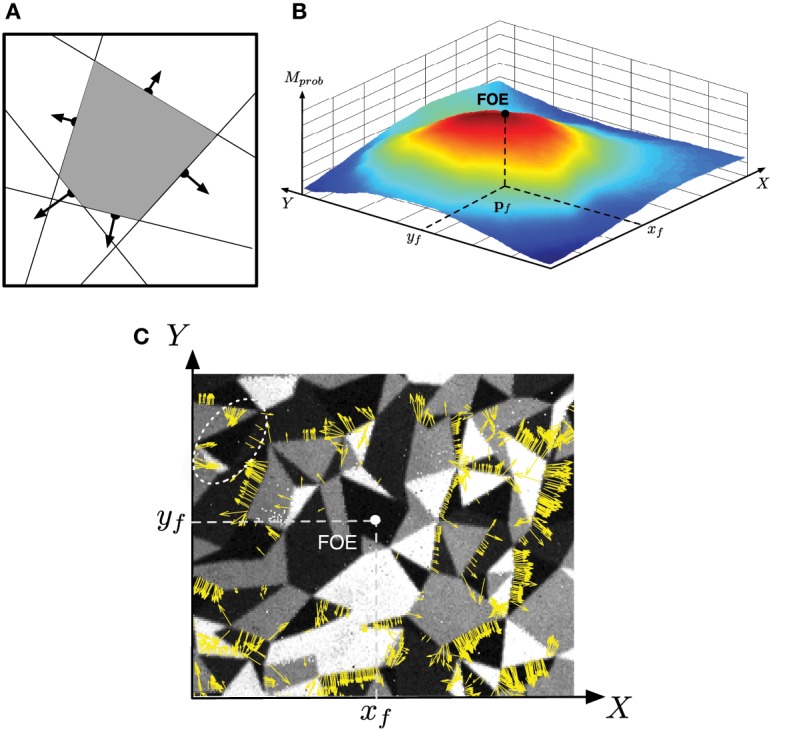
**Computation of the focus of expansion: (A) the focus of expansion lies under the normal flow, we can then vote for an area of the focal plane shown in (B) the FOE is the max of this area (C) Motion flow vectors obtained during a time period of Δ*t* = 10 ms and superimposed over a corresponding snapshot (realized using the PWM grayscale events; the viewed pattern is the same as used in Experiment 1, cf. Figure 7)**. Note that only the vectors with high amplitude are represented in order to enhance the readability of the Figure. Most of the motion flow vectors are diverging from the estimated FOE. The white ellipse in the up left corner shows a group of inconsistent motion flow vectors: they are probably due to a temporary noise micro-motion (vibration, unexpected roll-, pitch-, or yaw-motion).

The principle of the approach is described in Algorithm 1. We consider a probability map of the visual field, where each point represents the likelihood of the FOE to be located on the corresponding point in the field. Every flow provides an estimation of the location of the FOE in the visual field; indeed, because the visual flow is diverging from the FOE, it belongs to the negative semi-plane defined by the normal motion flow vector. So, for each incoming event, all the corresponding potential locations of the FOE are also computed (step 3 in Algorithm 1) and their likelihood is increased (step 4). Finding the location of the probability map with maximum value, the FOE is shifted toward this location (step 5)). This principe is illustrated in Figure 5A. The area with the maximum of probability is highlighted as the intersection of the negative semi-planes defined by the normal motion flow vectors. Finally, an exponential decreasing function is applied on the probability map; it allows updating the location of the FOE, giving more importance to the contributions provided by the most recent events and their associated flow.

**Algorithm 1:** **Computation of the Focus Of Expansion**.

**Require** *M*_*prob*_ ∈ ℝ^*m*^ × ℝ^*n*^ and *M*_*time*_ ∈ ℝ^*m*^ × ℝ^*n*^ (*M*_*prob*_ is the probability map and holds the likelihood for each spatial location and *M*_*time*_ the last time when its likelihood has been increased).
1: Initiate the matrices *M*_*prob*_ and *M*_*time*_ to 0
2: **for** every incoming *e*(**p**, *t*) at velocity **v_n_ do**
3: Determine all spatial locations **p***_i_* such as (**p**−**p_i_**)^*T*^.**v_n_** > 0
4: for all **p**_*i*_: *M*_*prob*_(**p**_*i*_) = *M_prob_*(**p**_*i*_) + 1 and *M*_*time*_(**p**_*i*_) = *t*_*i*_
5: ∀ **p**_*i*_ ∈ ℝ^*m*^ × ℝ^*n*^, update the probability map *M*_*prob*_(**p**_*i*_) = *M*_*prob*_(**p**_*i*_)$e^{-\frac{t_{i}-M_{time}(p_{i})}{\Delta t}}$
6: Find **p**_*f*_ = (*x_f,y__f_*)^*T*^ the spatial location of the maximum value of *M*_*prob*_ corresponding to the FOE location
7: **end for**

Figures 5B,C show real results obtained viewing a densely textured pattern (the same as used in Experiment 1, see Figure 7). Figure 5B shows the probability map defined as an accumulative table and the resulting FOE. The corresponding motion flow is given in Figure 5C; the normal motion vectors (with an amplitude superior than a threshold) computed in a time interval Δ*t* = 10 ms are represented as yellow arrows. Globally, the estimated FOE is consistent with the motion flow. However, some small groups of vectors (an example is surrounded by a white dotted ellipse) that seems converging, instead of diverging, to the FOE. Such flow events do not occur at the same time as the others; they are most probably generated by a temporary micro-motion (vibration, unexpected roll-, pitch- or yaw-motion). The cumulative process allows to filter such noise motions and to keep a FOE stable.

For an incoming event *e*(**p**, *t*) with a velocity vector **v**_n_, we can define the following algorithm to estimate the FOE:

## 4. Experimental results

**Figure 6:**
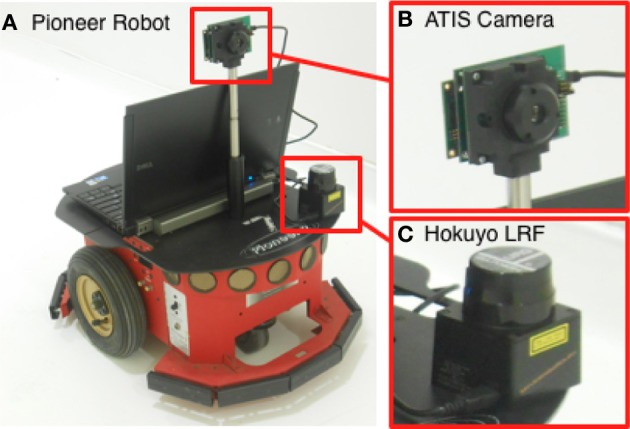
**Experimental setup: (A) the Pioneer 2, (B) the asynchronous event-based ATIS camera, (C) the Hokuyo laser range finder (LRF)**.

The method proposed in the previous sections is validated in the experimental setup illustrated in Figure 6. The neuromorphic camera is mounted on a Pioneer 2 robotic platform, equipped with a Hokuyo laser range finder (LRF) providing the actual distance between the platform and the obstacles. In experimental environment free of specular or transparent objects (as in the first proposed experiment), the TTC based on the LRF can be estimated using the Equation 1 and is used as ground truth measure against which the event-based TTC is benchmarked.

**Figure 7:**
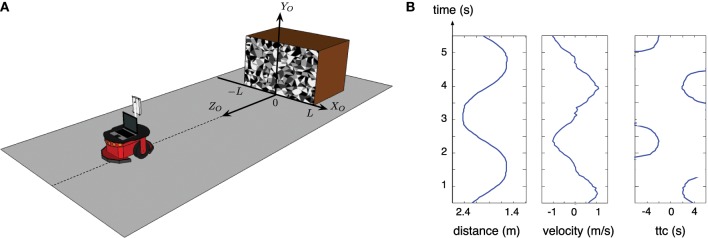
**First experiment: (A) setup and location of the coordinate system (*X_O_,Y_O_,Z_O_*) related to the obstacle; (B) distance between the robot and the obstacle, velocity of the robot and the relative estimated TTC over time are computed based on the odometer of the robot**. Only the TTC computed while the velocity of the robot is superior to 0.1 m/s is given, because it tends to infinity when velocity tends to 0.

**Figure 8:**
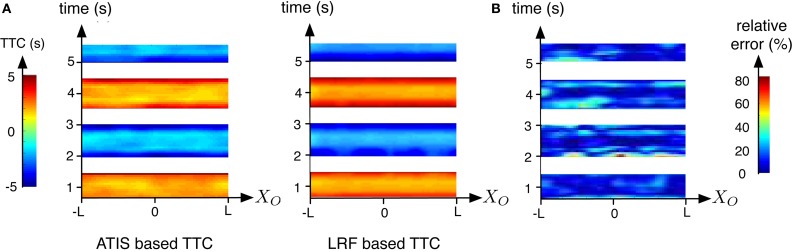
**Comparison of the results obtained while the robot is moving forward and backward in the direction of an obstacle**. Results are expressed related to time and the coordinates system of the obstacle. **(A)** TTC computed using the LRF (right) and the ATIS (left). **(B)** Relative errors between bothTTC estimations. illustrated using a color map, blue to red for increasing TTC

In the first experiment, the robot is moving forward and backward in the direction of a textured obstacle as shown in Figure 7, the corresponding TTC estimated by both sensors (LRF and ATIS) is shown in Figure 8A. The TTC is expressed in the coordinate system of the obstacle: the vertical axis corresponds to time and the horizontal axis to the size of the obstacle. The extremes (and the white parts of the plots) correspond to the changes of direction of the robot: when its speed tends to 0, the LRF based TTC tends to infinity and the vision based TTC cannot be computed because too few events are generated. In order to show comparable results, only the TTC obtained with a robot speed superior to 0.1 m/s are shown; under this value, the robot motion is relatively unstable, the robot tilting during the acceleration periods.

Figure 8B shows the relative error of the event-based TTC with respect of the ground truth calculated with the LRF TTC. The error is large during the phases of positive and negative accelerations of the robot. There are two potential explanations. The estimation of the speed of the robot based on the LRF is relatively inaccurate during the change of velocity. In addition, brutal changes of velocity could generate fast pitch motions which produce unstable FOE. Globally, more than the 60% of the relative errors are inferior to 20% showing that that the event-based approach is robust and accurate when the motion of the robot is stable.

**Figure 9:**
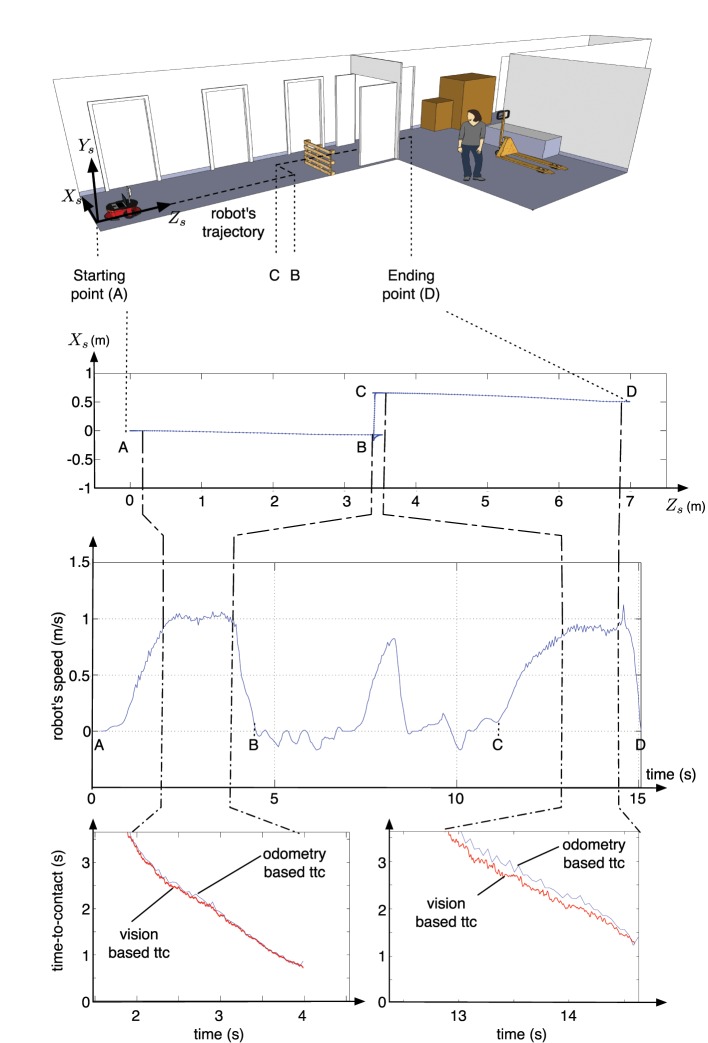
**Results of second experiment: the top Figure represents the trajectory followed by the the robot, on a schematic view of the warehouse, both middle Figures represents data collected from the odometer (the trajectory and the speed of the robot) and finally, the bottom Figures represent the time-to-contact estimated during the two time intervals during which the TTC is estimated; the red curves correspond to the TTC estimated from the neuromorphic camera's data, compared to an estimation of the ttc (blue curves) using the odometer's data and the knowledge of the obstacles' locations in the map**.

**Figure 10:**
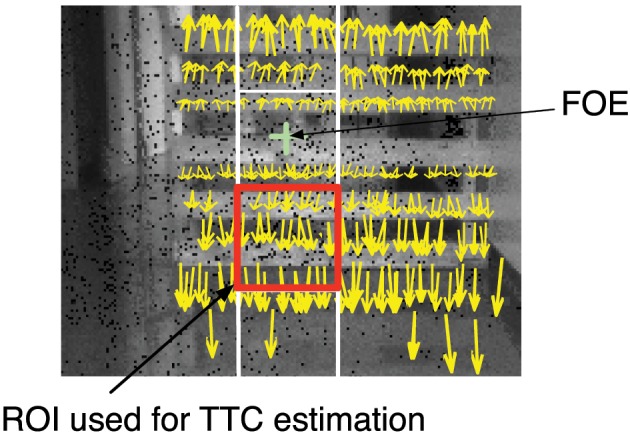
**TTC computation: the yellow arrows represent the motion flow vectors obtained during a time period of 1 ms**. These flow vectors are superimposed over a corresponding image of the scene (realized using the PWM grayscale events). In order to enhance the readability of the Figure, only 10% vectors with high strengths and orientations close to ±π/2 have been draw. The red square corresponds to the ROI where the measure of TTC is estimated.

In the second experiment, the robot moves along a corridor. In this conditions, multiple objects reflect the light from the LRF, that fails to detect obstacles, on the contrary the event-based algorithm succeeds in estimating the TTC relative to the obstacles. Figure 8 shows the robot's trajectory: during the first stage the robot navigates toward an obstacle (portion A-B of the trajectory). An avoidance maneuver is performed during portion B-C that leads the robot to continue its trajectory to enter the warehouse (portion C-D). The estimated TTC to the closest obstacle, is shown as red plots in Figure 9 and compared to the ground truth given by the odometer's data (in blue). It corresponds to the TTC collected in a region of interest of 60 × 60 pixels, matching with the closest obstacle. The image plane is segmented into four regions of interest (ROI) of 60 × 60 pixels (4 squares represented in the Figure 10) around the x-coordinate of the FOE. Only the normal flow vectors into the lower ROI, in which the activity, expressed as the number of events per second, is superior to a threshold (>5000 events/s), are considered, assuming that the closest obstacle is on the ground and so viewed in the bottom part of the vision field.

The low shift between them can be explained by the drift in odometer's data (especially after the avoidance maneuver) Everett (1995); Ge (2010); a difference of 0.5 m. has been observed between the real position of the robot and the odometer-based estimate of the ending location. This is an expected effect, as odometer always drifts in the same measured proportions Ivanjko et al. (2007). In addition, the estimations are slightly less precise once the robot is in the warehouse, where the poor environment with white walls without texture or objects produces less events and the computation degrades. This shows the robustness of the technique even in poorly textured.

All programs have been written in C++ under linux and run in real time. We estimated the average time per event spent to compute the Time-to-Contact: it is approximately 20 μs per event on the computer used in the experiments (Intel Core i7 at 2.40 GHz). When the robot is at its maximum speed, data stream acquired during 1 s is processed in 0.33 s. The estimation of the visual flow is the most computationally expensive task (>99% of the total computational cost), but could be easily run in parallel to further accelerate it.

The most significant result of this work is that the TTC can be processed at an unprecedented rate and with a low computational cost. The output frequency of our method reaches over 16 kHz, which is largely superior to the ones which can be expected from any other conventional cameras, limited by their frame-based acquisitions and processing load needed to process data.

## 5. Conclusions and perspectives

The use of vision based navigation using conventional frame-based cameras is impractical for the limited available resources usually embedded on autonomous robots. The corresponding large amount of data to process is not compatible with fast and reactive navigation commands, especially when parts of the processing are allocated to extract the useful information. Such computational requirements are out of the reach of most small robots. Additionally, the temporal resolution of frame-based cameras trades off with the quantity of data that need to be processed, posing limits on the robot's speed and computational demand. In this paper, we gave an example of a simple collision avoidance technique based on the estimation of the TTC by combining the use of an event-based vision sensor and a recent previously developed event-based optical flow. We showed that event-based techniques can solve vision tasks in a more efficient way than traditional approaches that are used to do, by means of complex and hungry algorithms.

One remarkable highlight of this work is how well the event-based optical flow presented in Benosman et al. (2014) helped in estimating the TTC. This is because we have ensured the preservation of the high temporal dynamics of the signal from its acquisition to its processing. The precise timing conveyed by the neuromorphic camera allows to process locally around each event for a low computational cost, whilst ensuring a precise computation of the visual motion flow and thus, of the TTC. The experiments carried out on a wheeled robotic platform support this statement, as the results are as reliable as the ones obtained with a laser range finder, at a much higher frequency. With event-based vision, the motion behavior of a robot could be controlled with a time delay far below the one that is inherent to the frame-based acquisition in conventional cameras.

The method described in this work stands on the constant velocity hypothesis since Equation 1 is a result derived from that assumption. for this reason, the normal to the edges velocity is sufficient for the TTC estimation. For more general motion, the proposed method should be modified by for example assuming the velocity to be constant only locally.

This work supports the observation that event-driven (bio-inspired) asynchronous sensing and computing are opening promising perspectives for autonomous robotic applications. The event-based approaches would allow small robots to avoid obstacles in natural environment with high speed that has never been achieved until now. Extending our approach to more complex scenarios than those exposed in this paper, and proposing a complete navigation system able to deal with motion or uncontrolled environment, requires to combine the visual information with other provided from top-down process and proprioceptive sensing, as for humans or animals.

### Conflict of interest statement

The authors declare that the research was conducted in the absence of any commercial or financial relationships that could be construed as a potential conflict of interest.
